# Fluoropyrimidine-induced cardiotoxicity: outcomes and safety of chemotherapy reintroduction in a retrospective cohort study

**DOI:** 10.1007/s00520-026-10531-2

**Published:** 2026-03-08

**Authors:** Océane Réa, Anissa Bouali, Michaël Serraille, Julien Péron, Claire Falandry, Justine Fort, Laurent François, Brahim Harbaoui, Pierre Lantelme, Pierre-Yves Courand

**Affiliations:** 1 Institut de Cardiologie, Hospices Civils de Lyon, Lyon, France; 2 Institut de Cancérologie, Hospices Civils de Lyon, Lyon, France; 3 Reshape Laboratory, U1290 INSERM, Lyon, France; 4 Institut du Vieillissement, Service de Gériatrie Croix-Rousse/Dugoujon, Hospices Civils de Lyon, Lyon, France; 5 Laboratoire CarMeN, INSERM U1060, Université Lyon 1/INRAE U1397/Hospices Civils de Lyon, Pierre-Bénite, France; 6 CREATIS, CNRS UMR5220, INSERM U1044, INSA-Lyon, Université Claude Bernard Lyon 1, Université de Lyon, Lyon, France; 7 Fédération de Cardiologie, Hôpital de La Croix-Rousse Et Lyon Sud, 103 Grande Rue de La Croix-Rousse, Lyon, 69004 France

**Keywords:** Fluoropyrimidine, 5-Fluorouracil, Cardiac toxicity, Myocardial infarction, Atrial fibrillation, Cancer

## Abstract

**Aims:**

Fluoropyrimidines, including 5-fluorouracil (5-FU) and capecitabine, remain foundational in the treatment of various cancers, despite their well-documented risk of cardiotoxicity. This study aimed to characterize cardiovascular events associated with fluoropyrimidine use, evaluate their management, and assess the prognostic impact of fluoropyrimidine reintroduction.

**Methods:**

We conducted a retrospective cohort study of patients admitted for cardiovascular events between January 2014 and October 2024 at Croix-Rousse and Lyon Sud University Hospitals (Hospices Civils de Lyon, France), who had received fluoropyrimidine-based chemotherapy within the preceding year.

**Results:**

Among 141 patients, the most frequent cardiovascular events were coronary artery disease (30.5%), atrial fibrillation (28.4%), and heart failure (19.9%). Post-event, three primary therapeutic strategies were implemented in the follow-up cohort (*n* = 114): fluoropyrimidine reintroduction (*n* = 54, 38.3%), switch to alternative chemotherapy (*n* = 25, 17.7%), and transition to palliative care (*n* = 36, 25.5%). Recurrent cardiotoxicity after reintroduction occurred in eight patients (14.8%), with only one recurrent coronary event. At 2-year follow-up, overall survival tended to be higher in the reintroduction group compared to the group of patients switching to an alternative chemotherapy (HR = 1.77 [0.92–3.42]; *p* = 0.088) and to palliative care (HR = 8.31 [4.67–14.79]; *p* < 0.001). No significant increase in unplanned hospitalizations was observed in the reintroduction group compared to the alternative chemotherapy group (HR = 1.48 [0.83–2.66]; *p* = 0.185).

**Conclusion:**

Our findings suggest that fluoropyrimidine reintroduction—guided by multidisciplinary evaluation, cardiovascular management, and close monitoring—appears to have a favorable benefit/risk balance for selected patients.

**Supplementary information:**

The online version contains supplementary material available at 10.1007/s00520-026-10531-2.

## Introduction

Fluoropyrimidines, including 5-fluorouracil (5-FU) and its oral prodrug capecitabine, are fundamental components of chemotherapy regimens for a wide range of cancers [1]. While generally well tolerated, these agents are associated with a well-documented spectrum of cardiotoxicity, ranging from myocardial ischemia and atrial fibrillation (AF) to heart failure (HF), myocarditis, and Takotsubo cardiomyopathy [2, 3]. Recent studies have highlighted a higher risk of heart failure, ischemic stroke, and chest pain [4]. Fluoropyrimidines are considered the second most cardiotoxic chemotherapeutic agents after anthracyclines [5]. Despite these risks, recent studies—particularly in patients with gastrointestinal cancers—have demonstrated a significant overall survival benefit associated with fluoropyrimidine-based chemotherapy, outweighing the potential cardiac complications [6]. Nevertheless, the risk stratification of fluoropyrimidine-induced cardiotoxicity remains an unresolved clinical challenge. Current guidelines recommend cardiovascular risk assessment using SCORE2 and advocate for coronary artery disease screening in high-risk patients, although the level of supporting evidence remains low [2]. Furthermore, no cardiovascular preventive strategy has yet demonstrated efficacy in reducing cardiotoxicity in randomized controlled trials. One of the most pressing unanswered questions is the optimal management of fluoropyrimidine therapy following a cardiac event, particularly acute coronary syndrome. Establishing a causal relationship between the cardiovascular event and fluoropyrimidine toxicity is often difficult due to the drug’s multiple potential mechanisms of injury. These include direct myocardial cell damage, endothelial dysfunction, and smooth muscle impairment—each of which can lead to both acute and long-term cardiovascular sequelae [7]. Decisions regarding the continuation or discontinuation of fluoropyrimidine therapy after such events require careful, individualized evaluation through a multidisciplinary collaboration between oncologists and cardiologists. However, data on fluoropyrimidine reintroduction after cardiotoxicity are limited. The reported recurrence of chest pain is highly variable from 10 to 50% of patients, even among those receiving anti-anginal therapy, following re-exposure to fluoropyrimidines [8, 9]. This high recurrence rate likely deters clinicians from resuming treatment, despite the potential oncologic benefit. In this context, further investigation is urgently needed to guide clinical decisions in the era of cardio-oncology. This study aimed to describe the clinical characteristics, management strategies, and outcomes of fluoropyrimidine-associated cardiotoxicity in a real-world cohort of cancer patients. Secondary objectives included evaluating the cardiac safety and prognostic implications of fluoropyrimidine reintroduction compared to alternative chemotherapy or palliative care.

## Methods

### Population

**Fig. 1:**
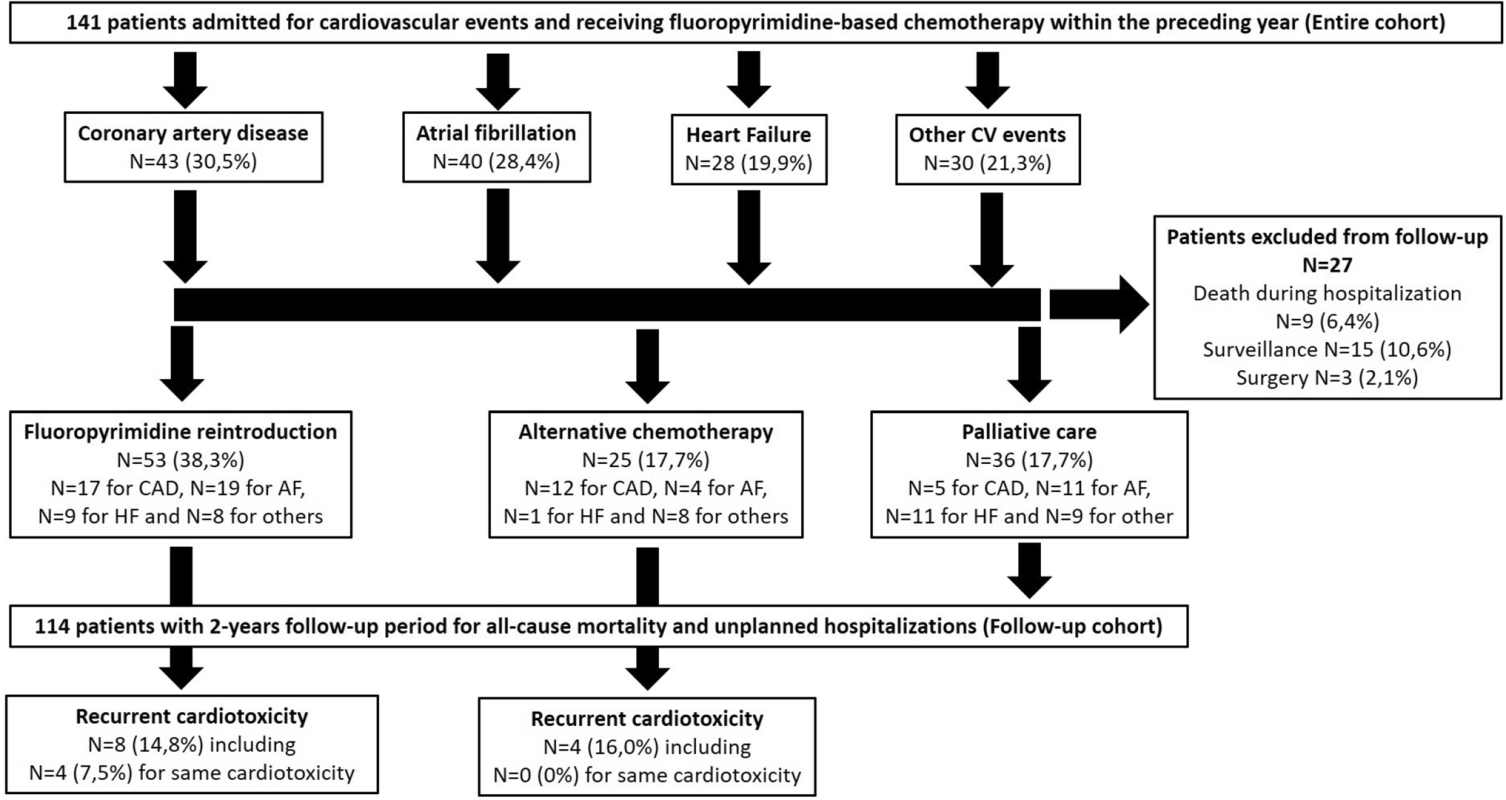
Flowchart including the entire cohort, cardiovascular events, and the follow-up cohort with or without fluoropyrimidine reintroduction

We conducted a retrospective cohort study at Croix-Rousse Hospital and Lyon Sud Hospital (Hospices Civils de Lyon, Lyon, France) between January 1, 2014, and October 30, 2024. Eligible patients were those hospitalized for a cardiac event and who had received fluoropyrimidine-based chemotherapy for cancer within the preceding 12 months. The entire cohort includes 141 patients who experienced a cardiovascular event during fluoropyrimidine treatment. The follow-up cohort after the event comprises 114 patients, of whom 53 underwent fluoropyrimidine reintroduction, 25 received alternative chemotherapy, and 36 were managed palliatively (Fig. 1). The French legislation on human subject research known as the Jardé law of 5 March 2012 and our study is classified in the non-interventional category. In accordance with French regulations, the study was approved by the institutional review board of the *Hospices Civils de Lyon*, France (CSE-HCL–IRB 00013204, August 14, 2025, number 25_5252, MR-004). In line with applicable legislation at the time of the study, all living participants or their next of kin if deceased were sent an information letter and given the opportunity to opt out of the use of their data for research purposes.

### Data collection

Clinical data were extracted from electronic medical records. The following variables were collected: demographic information (age, sex), cardiovascular risk factors and comorbidities (hypertension, diabetes, dyslipidemia, smoking status, prior cardiovascular disease), cancer type and stage, cancer treatment, details of the index cardiac diagnosis, cardiac investigations, management, and follow-up. Patients were classified into one of four categories based on their cardiovascular diagnosis: (1) coronary artery disease (CAD) including ST-elevation myocardial infarction (STEMI, acute coronary syndrome with persistent ST-segment elevation: new ST elevation at the J-point in at least two contiguous leads), non-ST-elevation myocardial infarction (NSTEMI, acute coronary syndrome without persistent ST-segment elevation: ECG may be normal or with ST depression or T wave changes) type 1 (characterized by atherosclerotic plaque rupture, ulceration, fissure, or erosion with resulting intraluminal thrombus in one or more coronary arteries, leading to decreased myocardial blood flow and/or distal embolization and subsequent myocardial necrosis) or type 2 (myocardial necrosis in which a condition other than coronary plaque instability causes an imbalance between myocardial oxygen supply and demand: hypotension, hypertension, tachyarrhythmias, bradyarrhythmias, anemia, hypoxemia, etc.), chronic coronary syndrome or suspected vasospastic angina; (2) AF defined as either a first episode or a recurrence; (3) HF as the primary diagnosis, sometimes in association with persistent AF; and (4) other diagnoses encompassing all remaining cardiac conditions not covered above. The time interval between fluoropyrimidine administration and the onset of cardiotoxicity was assessed using two distinct definitions: (1) the interval from the first fluoropyrimidine dose to the cardiovascular event, and (2) the interval from the most recent (last) fluoropyrimidine dose to the cardiovascular event.

### Statistical analyses

Quantitative variables were expressed as mean ± standard deviation (SD) or, for non-normally distributed data (assessed via Kolmogorov–Smirnov test and skewness), as medians with interquartile ranges [IQR]. Categorical variables were summarized as counts and percentages. Comparisons of continuous variables between groups were conducted using non-parametric tests (Mann–Whitney *U* or Jonckheere–Terpstra tests), as appropriate. Categorical variables were compared using Pearson’s *χ*^2^ test.

Survival analyses were conducted over a 2-year follow-up period from the index cardiac event. Outcomes of interest included all-cause mortality and unplanned hospitalization. These were estimated using the Kaplan–Meier method, with group comparisons performed via the log-rank test. Analyses were stratified by post-event management strategy: fluoropyrimidine reintroduction, switch to an alternative chemotherapy regimen, or transition to palliative care. Univariate Cox proportional hazards models were used to explore associations, and the proportional hazards assumption was tested for each covariate by including time-dependent interaction terms. All statistical analyses were performed using SPSS software, version 20.0.0 (SPSS Inc., Chicago, IL, USA). A two-sided *p*-value < 0.05 was considered statistically significant.

## Results

### Baseline characteristics and presentation

A total of 141 patients were included in the study (entire cohort). Their baseline characteristics prior to the cardiovascular event are summarized in Table 1. At the initiation of fluoropyrimidine therapy, more than one-third had colorectal cancer and 63 patients (44.7%) were treated for metastatic cancer. Five patients (3.5%) received capecitabine (oral), while the remaining received 5-FU via either a combination of bolus and continuous intravenous infusion (*n*=51), continuous intravenous infusion (*n*=64), or unknown administration route (*n*=21). Many patients received fluoropyrimidine in combination with other anticancer therapies, including platinum salts (*n*=97, 68.8%), irinotecan (*n*=34, 24.1%), taxanes (*n*=26, 18.4%), epidermal growth factor receptor inhibitors (*n*=11, 7.8%), vascular endothelial growth factor inhibitors (*n*=12, 8.5%), and immune checkpoint inhibitors (*n*=4, 2.8%). Cardiovascular toxicity occurred after a median of 4 [IQR 2–9] chemotherapy cycles. The median time from first fluoropyrimidine dose to onset of cardiotoxicity was 98 [29–209] days, and from the most recent (last) administration to the event was 15 [4–41] days (Figure S1 supplementary data, panels A and B respectively). The most frequent cardiotoxic events summarized in Fig. 1 were as follows: (1) CAD: *n*=43 (30.5%) including STEMI (*n*=10), type 1 NSTEMI (*n*=10), type 2 NSTEMI (*n*=8), chronic coronary syndrome (*n*=9), and presumed vasospastic angina (*n*=6); (2) AF: *n*=40 (28.4%) including *n*=13 with prior episodes of paroxysmal atrial fibrillation; (3) HF: *n*=28 (19.9%) including *n*=26 new-onset HF and *n*=2 acute decompensation of chronic HF; and (4) other events: arrhythmias (*n*=8, 5.6%), myocarditis (*n*=7, 5.0%), severe pericardial effusion (*n*=7, 5.0%), pulmonary embolism (*n*=3, 2.1%), Takotsubo cardiomyopathy (*n*=3, 2.1%), conduction disorders (*n*=1, 0.7%), and endocarditis (*n*=1, 0.7%). The median hospital stay was 3 [1–7] days. Cardiovascular presentations were compared across four diagnostic subgroups: CAD, AF, HF, and other cardiovascular events in Table S1 (Supplementary data). Significant differences were observed regarding the prevalence of hypertension (more common in CAD and “other” groups), prior AF history (more common in AF group), time from last fluoropyrimidine dose to toxicity (shorter in CAD), and concomitant use of immune checkpoint inhibitors (more frequent in “other” toxicities, especially myocarditis).

**Table 1:** Baseline characteristics of the entire cohort

	Entire cohort (*N* = 141)
Age (years)	69 [59–78]
Gender (male)	96 (68.1)
Cardiovascular history and risk factors	
Diabetes	26 (18.4)
Hypertension	71 (50.4)
Dyslipidemia	41 (29.1)
Tobacco never/former/current	61 (43.3)/49 (34.8)/31 (22.0)
Heart failure	12 (8.5)
Coronary artery disease	18 (12.8)
Atrial fibrillation	25 (17.7)
Valvular heart disease	6 (4.3)
Metastatic cancer	63 (44.7)
Subtype of cancer	
Colorectal	56 (39.7)
ENT	27 (19.1)
Gastric	18 (12.8)
Pancreas	15 (10.6)
Esophagus	15 (10.6)
Breast	4 (2.8)
Other	6 (4.3)

The data are *n* (%)

### Cardiovascular investigation and therapeutic management

Cardiovascular workup and management strategies are summarized in Table 2. Among the seven patients who developed myocarditis, four were receiving concomitant treatment with immune checkpoint inhibitors (three with nivolumab and one with pembrolizumab). The diagnosis was confirmed by cardiac MRI for all of them. Those treated with immune checkpoint inhibitors received intravenous corticosteroid treatment followed by an oral formulation. All seven patients hospitalized for pericarditis presented with pericardial effusion and underwent percutaneous drainage during their hospital stay. All of them had a large effusion (> 20 mm) and symptoms related to pericardial effusion, including cardiogenic shock in one case and dyspnea in six. In the CAD subgroup, coronary angiography was performed in 83% of patients, with significant coronary lesions found in 69.2%. Among the remaining patients (*n* = 12), two had a negative ergonovine test using intracoronary injection. Percutaneous coronary intervention (PCI) was performed in 20 patients from the CAD group and in two patients from other groups. None underwent coronary artery bypass grafting. Detailed CAD management is available in Table S2 (supplementary material). In patients with additional transthoracic echocardiography after the index cardiovascular event (*n* = 96), LVEF increased significantly at 60% [50–63] (*p* = 0.006). Following the cardiotoxic event, multidisciplinary team decisions were as follows: reintroduction of fluoropyrimidine in 54 patients (38.3%), switch to a non-fluoropyrimidine chemotherapy in 25 (17.7%), transition to palliative care in 36 (25.5%), surveillance only in 15 (10.6%), and surgery after neoadjuvant chemotherapy in three (2.1%). Nine patients (6.4%) died during the index hospitalization (Fig. 1). The subsequent analyses focused on the three primary therapeutic strategies in the follow-up cohort (Fig. 1): fluoropyrimidine reintroduction, switch to a non-fluoropyrimidine chemotherapy, and palliative care. Characteristics of the follow-up cohort and these subgroups are shown in Table 3. Significant differences were noted in cancer type, metastatic status, and type of cardiotoxicity. Particularly, we observed a significant less frequency of metastatic status in the group switch to a non-fluoropyrimidine chemotherapy. The second-line chemotherapy regimens are listed in Table S3 (supplementary material). Conditions for reintroduction varied depending on the cardiac event. Nine patients were re-challenged with fluoropyrimidine under cardiology unit monitoring following a coronary event (two STEMI, three chronic coronary syndrome, four vasospastic angina). The remaining reintroductions occurred in oncology wards (conventional or day hospital settings) or at home (Table 4).

**Table 2:** Cardiovascular investigation at admission and management of cardiovascular toxicities

Toxicities	Entire cohort	Coronary artery disease	Atrial fibrillation	Heart failure	Other	*p* value
*N* (%)	141 (100)	43 (30.5)	40 (28.4)	28 (19.9)	30 (21.3)	
ECG characteristics						
Atrial fibrillation	54 (38.3)	5 (11.6)	40 (100.0)	6 (21.4)	3 (10)	< 0.001
Isolated abnormal T waves	28 (19.9)	15 (34.9)	0 (0)	5 (17.9)	8 (26.7)	0.001
ST-segment elevation	13 (9.2)	11 (25.6)	0 (0)	0 (0)	2 (6.7)	< 0.001
Transthoracic echocardiography data						
LVEF (%)	55 [50–60]	55 [50–60]	55 [50–55]	50 [30–55]	55 [45–65]	0.081
Significant valvular heart disease	4 (2.8)	2 (4.7)	1 (2.5)	1 (3.6)	0 (0)	0.691
Significant pericardial effusion	8 (5.7)	0 (0)	1 (2.5)	0 (0)	7 (23.3)	< 0.001
Coronary angiogram, *n *(%)	55 (37.7)	39 (83.0)	0 (0)	4 (14.3)	12 (38.7)	< 0.001
No significant coronary artery stenoses, *n* (%)	21 (38.2)	12 (30.8)	0 (0)	2 (50.0)	7 (58.3)	
1-vessel disease, *n* (%)	20 (36.4)	13 (33.3)	0 (0)	2 (50.0)	5 (41.7)	
2-vessel disease, *n* (%)	7 (12.7)	7 (17.9)	0 (0)	0 (0)	0 (0)	
3-vessel disease, *n* (%)	7 (12.7)	7 (17.9)	0 (0)	0 (0)	0 (0)	
PCI, *n* (%)	22 (15.6)	20 (46.5)	0 (0)	1 (3.6)	1 (3.3)	< 0.001
Pericardiocentesis, *n *(%)	7 (5.0)	0 (0)	0 (0)	0 (0)	7 (23.3)	< 0.001
Medical treatment						
Anticoagulant, *n* (%)	41 (29.1)	6 (14.0)	22 (55.0)	5 (17.9)	8 (26.7)	< 0.001
Aspirin, *n* (%)	37 (26.2)	26 (60.5)	3 (7.5)	2 (7.1)	6 (20.0)	< 0.001
P2Y12 inhibitors, *n* (%)	24 (17.0)	21 (48.8)	0 (0)	1 (3.6)	2 (6.7)	< 0.001
Beta-blockers, *n* (%)	52 (36.9)	16 (37.2)	15 (37.5)	7 (25.0)	14 (46.7)	0.401
CCB dihydropyridine, *n *(%)	12 (8.5)	5 (11.6)	4 (10.0)	3 (10.7)	0 (0)	0.306
CCB benzothiazepine,* n* (%)	9 (6.4)	8 (18.6)	0 (0)	1 (3.6)	0 (0)	0.001
Nitrates, *n* (%)	13 (9.2)	12 (27.9)	0 (0)	0 (0)	1 (3.3)	< 0.001
Statins, *n* (%)	38 (27.0)	23 (53.5)	8 (20.0)	3 (10.7)	4 (13.3)	< 0.001

The data are *n* (%) or median [interquartile range, *IQR*]

*LVEF* left ventricular ejection fraction, *PCI* percutaneous coronary intervention, *CCB* calcium channel blockers

**Table 3:** Comparison between subgroups according to therapeutic strategy in the follow-up cohort (*N* = 114)

	Follow-up cohort	Fluoropyrimidine reintroduction	Second-line chemotherapy	Palliative care	*p* value
*N* (%)	*N* = 114	*N *= 53	*N* = 25	*N* = 36	-
Age	68 [58–76]	68 [59–77]	63 [55–74]	69 [59–76]	0.728
Male	77 (67.5)	33 (62.3)	19 (76.0)	25 (69.4)	0.461
Diabetes	21 (18.4)	11 (20.8)	4 (16.0)	6 (16.7)	0.834
Hypertension	61 (53.5)	29 (54.7)	15 (60.0)	17 (47.2)	0.599
Dyslipidemia	33 (28.9)	14 (26.4)	10 (40.0)	9 (25.0)	0.382
Tobacco never/former/current	52 (45.6)/36 (31.6)/26 (22.8)	27 (50.9)/17 (32.1)/9 (17.0)	6 (24.0)/8 (32.0)/11 (44.0)	19 (52.8)/11 (30.6)/6 (16.7)	0.047
History of heart failure	11 (9.6)	5 (9.4)	3 (12.0)	3 (8.3)	0.890
History of coronary artery disease	13 (11.4)	9 (17.0)	4 (16.0)	0 (0)	0.034
History of atrial fibrillation	21 (18.4)	10 (18.9)	4 (16.0)	7 (19.4)	0.937
History of valvular heart disease	5 (4.4)	3 (5.7)	2 (8.0)	0 (0)	0.268
Metastatic cancer	57 (50.0)	27 (50.9)	7 (28.0)	23 (63.9)	0.022
Subtype of cancer					0.003
Colorectal	45 (39.5)	23 (43.4)	9 (36.0)	13 (36.1)	
ENT	20 (17.5)	5 (9.4)	12 (48.0)	3 (8.3)	
Gastric	14 (12.5)	8 (15.1)	1 (4.0)	5 (13.9)	
Pancreas	14 (12.5)	5 (9.4)	1 (4.0)	8 (22.2)	
Esophagus	12 (10.5)	6 (11.3)	1 (4.0)	5 (13.9)	
Breast	4 (3.5)	4 (7.5)	0 (0)	0 (0)	
Other	5 (4.4)	2 (3.8)	1 (4.0)	2 (5.6)	
Cardiac toxicity event					0.012
Coronary artery disease	34 (29.8)	17 (32.1)	12 (48.0)	5 (13.9)	
Atrial fibrillation	34 (29.8)	19 (35.8)	4 (16.0)	11 (30.6)	
Heart failure	21 (18.4)	9 (17.0)	1 (4.0)	11 (30.6)	
Other	25 (21.9)	8 (15.1)	8 (32.0)	9 (25.0)

**Table 4:** Management after 5-FU cardiotoxicity

Toxicities	Entire cohort	Coronary artery disease	Atrial fibrillation	Heart failure	Other	*p* value
Chemotherapy decision						0.012
Fluoropyrimidine reintroduction	53 (37.6)	17 (39.5)	19 (47.5)	9 (32.1)	8 (26.7)	
Switch to another chemotherapy	25 (17.7)	12 (25.6)	4 (10.0)	1 (3.6)	8 (26.7)	
Palliative care	36 (25.5)	5 (11.6)	11 (27.5)	11 (39.3)	9 (30.0)	
Other issues						
Surgery after neoadjuvant chemotherapy	3 (2.1)	1 (2.3)	2 (5.0)	0 (0)	0 (0)	
Surveillance	15 (10.6)	4 (9.3)	3 (7.5)	5 (17.9)	3 (10.0)	
Death during hospitalization	9 (6.4)	4 (9.3)	1 (2.5)	2 (7.1)	2 (6.7)	
Place of fluoropyrimidine reintroduction	53	17	19	9	8	
Cardiology unit	8 (15.1)	8 (47.1)	0 (0)	0 (0)	0 (0)	0.001
Oncology wards	20 (37.7)	5 (29.4)	8 (42.1)	4 (44.4)	3 (37.5)	
Day oncology wards	23 (43.4)	2 (11.8)	11 (57.9)	5 (55.6)	5 (62.5)	
At home	2 (3.8)	2 (11.8)	0 (0)	0 (0)	0 (0)	
Follow-up after reintroduction of fluoropyrimidine	53	17	19	9	8	
Repeated hospitalization for the same cardio-toxicity	4 (7.5)	1 (5.9)	1 (5.3)	1 (11.1)	1 (12.5)	0.884
Repeated hospitalization for any cardio-toxicity	8 (15.1)	2 (11.8)	4 (21.1)	1 (11.1)	1 (12.5)	0.941
Follow-up after switch to non-fluoropyrimidine chemotherapy	25	12	4	1	8	
Repeated hospitalization for the same cardio-toxicity	0 (0)	0 (0)	0 (0)	0 (0)	0 (0)	-
Repeated hospitalization for any cardio-toxicity	4 (16.0)	3 (25.0)	0 (0)	0 (0)	1 (12.5)	0.818

### Follow-up and survival in the cohort

**Fig. 2:**
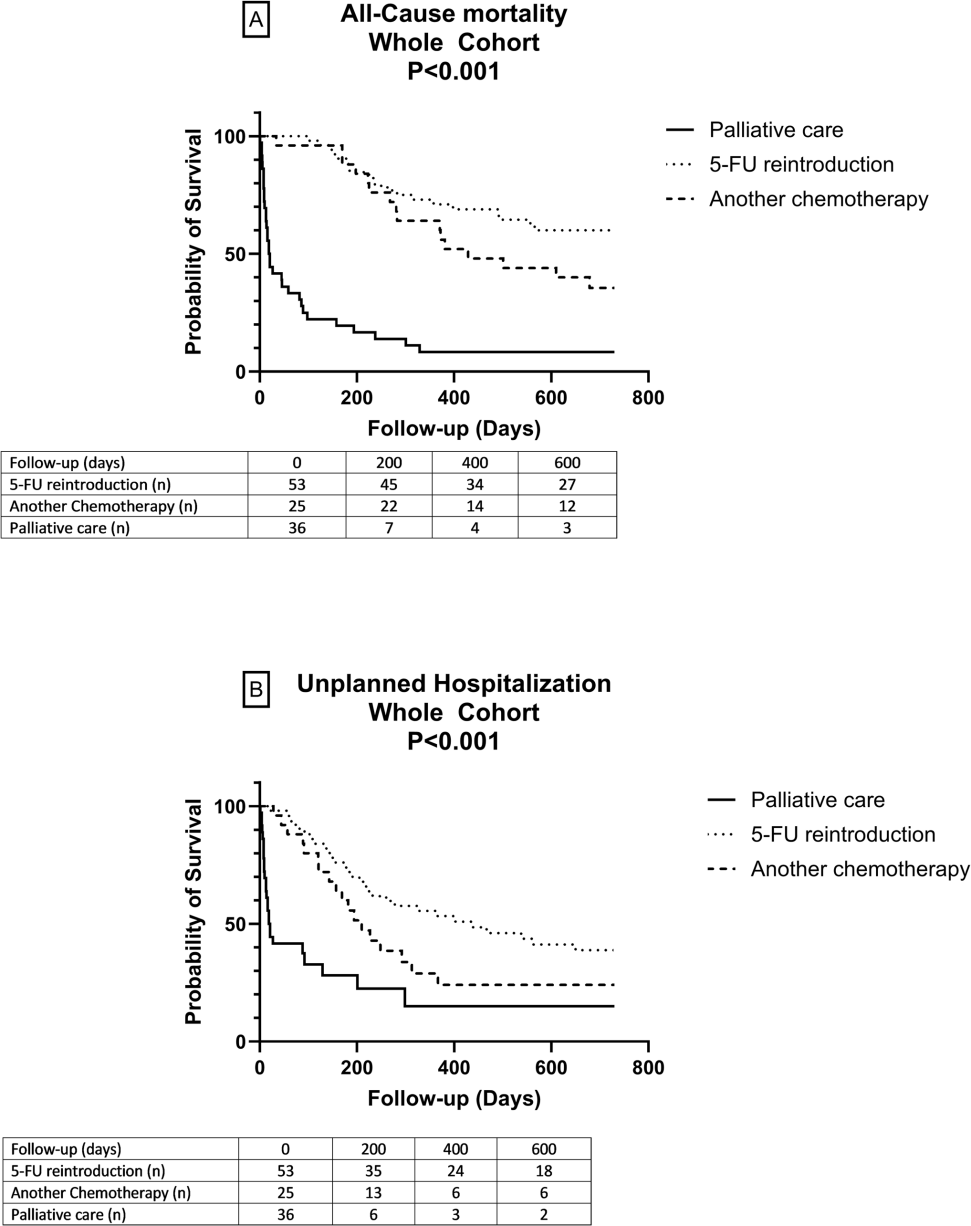
Kaplan–Meier curves in the follow-up cohort for all-cause mortality (**A**) and for unplanned hospitalization (**B**). *p* value for log-rank test

Survival analysis excluded patients who underwent surgery after neoadjuvant chemotherapy, were under surveillance following adjuvant therapy, or died during the initial hospitalization (Fig. 1). Focusing on the follow-up cohort (*n* = 114), all-cause mortalities were as follows 20 deaths (37.7%) in the fluoropyrimidine reintroduction group, 16 deaths (64.0%) in the switch to a non-fluoropyrimidine chemotherapy group, 33 deaths (91.7%) in the palliative care group, and 10 deaths (37.0%) in the remaining subgroups. Median survival were 20 days in the palliative care group, 429 days in the second-line chemotherapy group, and not reached in the fluoropyrimidine reintroduction group after the 2-year follow-up. Kaplan–Meier curves for all-cause mortality are shown in Fig. 2A. At 2-year follow-up in univariate Cox regression analysis, overall survival tended to be higher in the reintroduction group compared to the group of patients switching to an alternative chemotherapy (HR = 1.77 [0.92–3.42]; *p* = 0.088) and to palliative care (HR = 8.31 [4.67–14.79]; *p* < 0.001).

Unplanned hospitalizations occurred in the following: 31 patients (58.5%) in the fluoropyrimidine reintroduction group, 18 (72.0%) in the switch to a non-fluoropyrimidine chemotherapy group, 26 (72.2%) in the palliative care group, and 10 (37.0%) in other subgroups. Among the 54 patients who resumed fluoropyrimidine, four (7.4%) were re-hospitalized for the same cardiac issue, and eight (14.8%) for either recurrence or a different cardiotoxicity. There was no significant difference in recurrence rates across types of cardiotoxicity (Table 4). Notably, only one patient (1.9%) experienced a second coronary event, which followed the discontinuation of antiplatelet therapy due to a hemorrhagic complication. In the second-line chemotherapy group, four patients (16.0%) were re-hospitalized for new cardiovascular events. Kaplan–Meier curves for unplanned hospitalization are presented in Fig. 2B. A trend toward improved outcomes was observed in the fluoropyrimidine reintroduction group. At 2-year follow-up in univariate Cox regression analysis, unplanned hospitalizations tended to be lower in the reintroduction group compared to the group of patients switching to an alternative chemotherapy (HR = 1.48 [0.83–2.66]; *p* = 0.185) and to palliative care (HR = 3.83 [2.22–6.60]; *p* < 0.001).

## Discussion

**Fig. 3:**
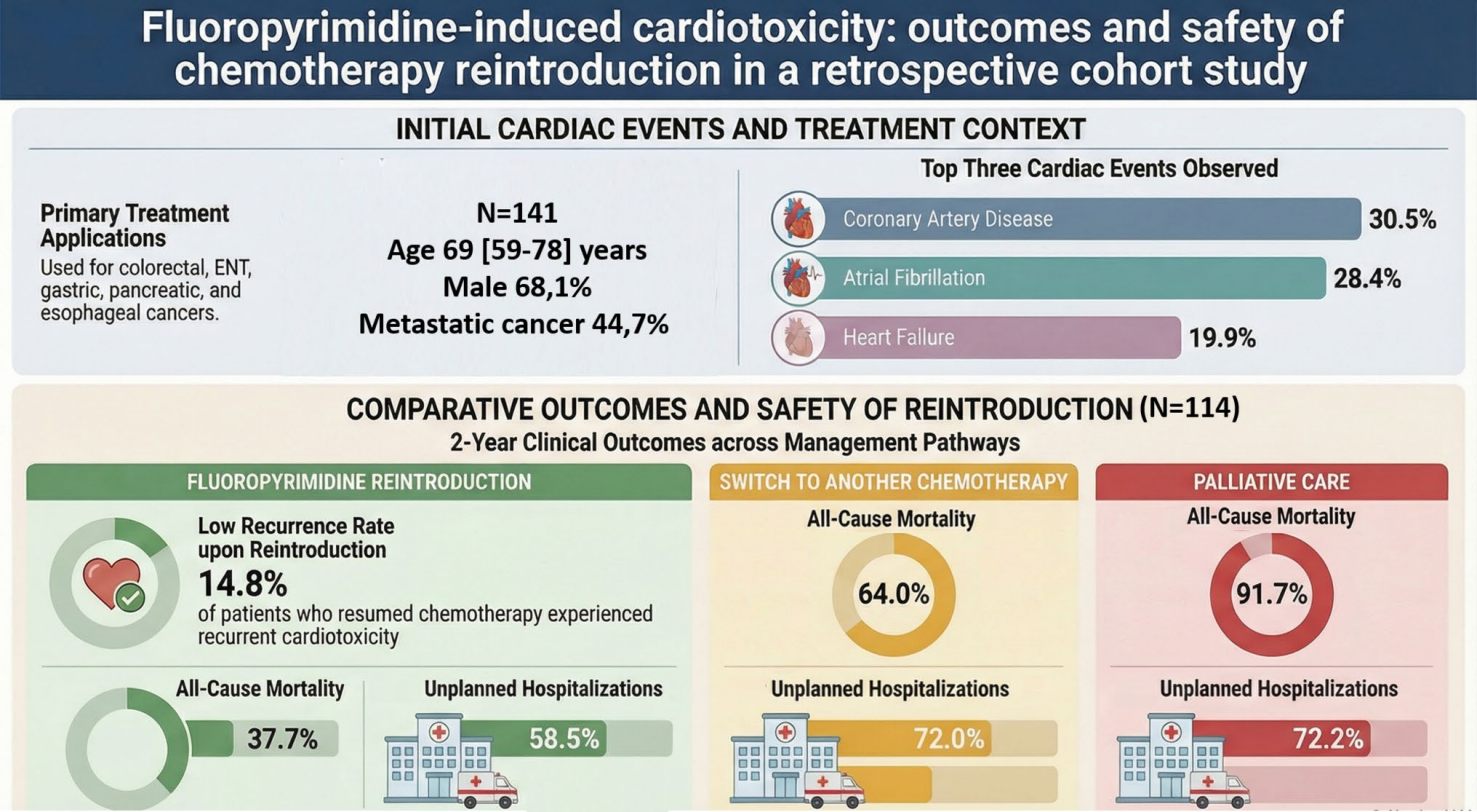
Central figure

This cohort study aimed to characterize fluoropyrimidine-associated cardiotoxicity and evaluate the prognosis of patients who underwent fluoropyrimidine reintroduction after a cardiovascular event, compared to those treated with a switch to a non-fluoropyrimidine chemotherapy or receiving palliative care. Reassuringly, no significant increase in unplanned hospitalizations was observed after fluoropyrimidine reintroduction, suggesting that a carefully managed rechallenge may be safe. It is worth noting that patients in the reintroduction group had a higher proportion of metastatic disease than those switching to a non-fluoropyrimidine chemotherapy, which could potentially bias survival with a less favorable expected outcome in the fluoropyrimidine reintroduction group. Among the 141 patients included between 2014 and 2024 in two academic centers, the most common cardiovascular events were CAD (30.5%), AF (28.4%), and HF (19.9%). Fluoropyrimidine reintroduction was attempted in 54 patients (38.3%), with a recurrence of cardiotoxicity in eight cases (14.8%), including only one recurrent coronary event. At 2 years, overall survival was higher in the reintroduction group compared to alternative chemotherapy and significantly better than in the palliative care group, although statistical significance was not reached when comparing with second-line chemotherapy (Fig. 3).

Fluoropyrimidine-induced cardiotoxicity is a well-documented adverse effect with highly variable presentation reflecting a complex and multifactorial pathophysiology. Coronary vasospasm and acute coronary syndromes are most commonly reported representing half of cases [10]. Several mechanisms have been proposed, including vascular endothelial damage followed by coagulation, ischemia secondary to coronary artery spasm, direct toxicity on the myocardium, and thrombogenicity [11]. Recently, capecitabine and 5-fluoropyrimidine have been associated in large registry-based case–control studies with a twofold increase in risk of myocardial infarction at 6 months [12, 13]. In a different design including only cardiovascular events, we observed in our study that coronary artery events account for nearly one-third of cases. However, the spectrum of toxicity extends beyond ischemic events. Our series included cases of myocarditis, pericarditis, conduction abnormalities, and Takotsubo cardiomyopathy, reflecting mechanisms such as direct myocardial injury, microvascular dysfunction, and inflammatory processes—mechanisms previously described in preclinical and case-report data. Despite these risks, the oncologic benefit of 5-FU often outweighs its cardiovascular toxicity. In a large cohort study of over 100,000 digestive cancer patients, Abiodun et al. showed that 5-FU use, while associated with slightly increased cardiovascular complications, significantly improved 1-year survival (41.9% vs. 49.6%) [6].

There is, however, a lack of standardized protocols for managing fluoropyrimidine-induced cardiotoxicity, particularly regarding reintroduction. Current ESC guidelines acknowledge the risk but offer minimal practical guidance. Clinical decision-making often relies on multidisciplinary collaboration between oncologists and cardiologists. Recurrence rates upon reintroduction remain poorly defined. Some studies report chest pain recurrence rates of up to 50%, even with prophylactic anti-anginal therapy [8]. In contrast, our data suggest that with rigorous patient selection, close cardiac monitoring, and individualized preventive strategies—including vasodilator therapy in suspected vasospasm and optimized anti-ischemic treatment—recurrence rates can be significantly reduced (< 15%) as also demonstrated in the CHECKPOINT trial [9]. Notably, most challenging reintroductions in our study occurred in cardiology units with continuous ECG monitoring, and in cases of confirmed coronary lesions, patients underwent revascularization before chemotherapy was resumed. The chemotherapy regimens themselves were largely maintained without modification and prescribed in coordination with oncology teams including physicians and nurses. The tolerance was generally good. Interestingly, two patients switched from bolus to continuous infusion with no recurrent events, challenging the assumption that continuous infusion always carries higher risk, likely due to prolonged plasma exposure [14–16]. Our findings suggest that under controlled conditions, even initial regimens may be safely reintroduced.

Despite efforts to identify predictors of fluoropyrimidine cardiotoxicity, no validated risk stratification tool currently exists. Several studies did not find an association of traditional cardiovascular risk factors with higher rates of fluoropyrimidine-induced cardiotoxicities [9, 17, 18]. Particularly, Lyhne et al. did not find that subclinical CAD using coronary artery calcium score predicts the risk of cardiotoxicity in cancer patients receiving fluoropyrimidine treatment. [19] While dihydropyrimidine dehydrogenase (DPD) deficiency is known to increase the risk of hematologic and gastrointestinal toxicity, its link to cardiotoxicity remains controversial [14, 20, 21]. In our cohort, five patients had partial DPD deficiency, with no case of complete deficiency. Due to missing data, we could not explore potential associations between DPD status and cardiac events. Prospective studies are needed to investigate this and other possible predictive markers.

### Study limitations

This study has several limitations. First, its retrospective nature may have led to selection and information bias, including missing data and potential underreporting of milder or undocumented cardiac events. Second, the study was conducted in two tertiary centers, which may limit generalizability. Third, the small sample size in some subgroups (particularly second-line chemotherapy and palliative care) reduces statistical power, and survival analyses were performed without adjustment for confounders such as age, comorbidities, tumor burden, or performance status. Lastly, heterogeneity in reintroduction protocols—including differences in monitoring, prophylactic strategies, and chemotherapy regimens—reflects real-world practice but limits reproducibility. These findings highlight the urgent need for standardized cardio-oncology pathways and protocols.

In conclusion, this study underscores the value of a multidisciplinary approach in managing fluoropyrimidine cardiotoxicity and suggests that fluoropyrimidine reintroduction, when conducted under strict cardiac surveillance and with appropriate prophylactic measures, may be both safe and beneficial in selected patients. While further prospective and multicentric studies are needed to establish clear guidelines, our findings support a proactive strategy rather than systematic discontinuation of an effective chemotherapy agent.

## Supplementary information

Below is the link to the electronic supplementary material.

ESM 1 (DOCX 71.7 KB)

## Data Availability

The datasets generated and/or analyzed during the current study are not publicly available due to but are available from the corresponding author on reasonable request for reviewing process.

## Declarations

### Generative artificial intelligence

The central figure has been generated with Google Notebook LM then modified by the authors.
