# Supplementary material for: Fluoropyrimidine-induced cardiotoxicity: outcomes and safety of chemotherapy reintroduction in a retrospective cohort study
Source: Support Care Cancer. 2026 Mar 8;34(4):293. doi: 10.1007/s00520-026-10531-2 (PMC12967614; doi:10.1007/s00520-026-10531-2)
Supplement: Supplementary file 1 — (DOCX 71.7 KB) [file 520_2026_10531_MOESM1_ESM.docx]

Supplementary data

Table S1. Cardiovascular toxicities presentation comparisons

| Toxicities | Coronary artery disease | Atrial fibrillation | Heart Failure | Other | P value |
| --- | --- | --- | --- | --- | --- |
| N (%) | 43 (30.5) | 40 (28.4) | 28 (19.9) | 30 (21.3) | - |
| Age | 70 [59-83] | 71 [59-76] | 64 [56-76] | 69 [59-80] | 0.307 |
| Male | 27 (62.8) | 30 (75.0) | 18 (64.3) | 21 (70.0) | 0.643 |
| Diabetes | 10 (23.3) | 8 (20.0) | 5 (17.9) | 3 (10.0) | 0.541 |
| Hypertension | 27 (62.8) | 15 (37.5) | 10 (35.7) | 19 (63.3) | 0.021 |
| Dyslipidemia | 10 (23.3) | 13 (32.5) | 7 (25.0) | 11 (36.7) | 0.573 |
| Tobacco never / former / current | 18 (41.9) / 13 (30.2) / 12 (27.9) | 15 (37.5) / 17 (42.5) / 8 (20.0) | 11 (39.3) / 12 (42.9) / 5 (17.9) | 17 (56.7) / 7 (23.3) / 6 (20.0) | 0.506 |
| History of HF | 3 (7.0) | 5 (12.5) | 2 (7.1) | 2 (6.7) | 0.766 |
| History of CAD | 10 (23.3) | 3 (7.5) | 2 (7.1) | 3 (10.0) | 0.100 |
| History of AF | 2 (4.7) | 13 (32.5) | 6 (21.4) | 4 (13.3) | 0.009 |
| History of VHD | 3 (7.0) | 1 (2.5) | 0 (0) | 2 (6.7) | 0.431 |
| Number of chemotherapy courses before cardiovascular toxicities | 4 [2-8] | 5 [3-8] | 5 [1-12] | 4 [3-11] | 0.875 |
| Time between the first fluoropyrimidine dose and cardiotoxicity (days) | 55 [12-204] | 110 [59-191] | 116 [23-276] | 92 [54-214] | 0.264 |
| Time between the most recent fluoropyrimidine dose and cardiotoxicity (days) | 8 [1-27] | 19 [6-39] | 23 [3-45] | 17 [6-44] | 0.046 |
| Capecitabine / 5-FU administration N (%) | 4 (9.3) / 39 (90.7) | 1 (2.5) / 39 (97.5) | 0 (0) / 28 (100.0) | 0 (0) / 30 (100.0) | 0.093 |
| Hospital stay (days) | 2 [1-4] | 4 [1-10] | 4 [1-9] | 3 [1-7] | 0.324 |
| Other anticancer drugs |  |  |  |  |  |
| Platinum salt, N (%) | 30 (69.8) | 29 (72.5) | 20 (71.4) | 18 (60.0) | 0.695 |
| Taxane, N (%) | 9 (20.9) | 8 (20.0) | 4 (14.3) | 5 (16.7) | 0.890 |
| ICI, N (%) | 0 (0) | 0 (0) | 0 (0) | 4 (13.3) | 0.002 |
| EGFR inhibitors, N (%) | 5 (11.6) | 2 (5.0) | 1 (3.6) | 3 (10.0) | 0.530 |
| VEGF inhibitors, N (%) | 3 (7.0) | 2 (5.0) | 5 (17.9) | 2 (6.7) | 0.258 |
| Irinotecan, N (%) | 7 (16.3) | 10 (25.0) | 10 (35.7) | 7 (23.3) | 0.226 |

The data are n (%) or median [interquartile range, IQR].

ICI: Immune checkpoint inhibitors, EGFR: epidermal growth factor receptor inhibitors, VEGF: vascular endothelial growth factor, 5-FU: 5-fluorouracil; AF: atrial fibrillation; HF: heart failure; CAD: coronary artery disease; VHD: valvular heart disease

Table S2. Detailed treatment in the coronary artery disease subgroup

| Toxicities | STEMI | Type 1  NSTEMI | Type 2  NSTEMI | Presumed  vasospasm | Chronic coronary syndrome |
| --- | --- | --- | --- | --- | --- |
| N (%) | 10 (23.3) | 10 (23.3) | 8 (18.6) | 6 (14.0) | 9 (20.9) |
| PCI, n (%) | 9 (90.0) | 7 (70.0) | 0 (0) | 0 (0) | 4 (44.4) |
| Medical treatment |  |  |  |  |  |
| Anticoagulant, n (%) | 3 (30.0) | 0 (0) | 0 (0) | 0 (0) | 3 (33.3) |
| Aspirin, n (%) | 9 (90.0) | 10 (100.0) | 1 (12.5) | 0 (0) | 6 (66.7) |
| P2Y12 inhibitors, n (%) | 9 (90.0) | 7 (70.0) | 1 (12.5) | 0 (0) | 4 (44.4) |
| Beta-blockers, n (%) | 5 (50.0) | 7 (70.0) | 0 (0) | 0 (0) | 4 (44.4) |
| CCB dihydropyridine, n (%) | 0 (0) | 2 (20.0) | 0 (0) | 2 (33.3) | 1 (16.7) |
| CCB benzothiazepine, n (%) | 1 (10.0) | 1 (10.0) | 0 (0) | 3 (50.0) | 3 (33.3) |
| Nitrates, n (%) | 4 (40.0) | 1 (10.0) | 0 (0) | 2 (33.3) | 5 (55.6) |
| Statins, n (%) | 6 (60.0) | 10 (100.0) | 1 (12.5) | 0 (0) | 6 (66.7) |

CCB: calcium channel blocker; PCI: percutaneous coronary intervention

Table S3. Second line of chemotherapy after cardiovascular toxicity with 5-fluoro-uracile (N=25)

| Chemotherapy | Number of patients |
| --- | --- |
| Carboplatine | 1 |
| Cetuximab | 2 |
| Cetuximab and irinotecan | 1 |
| Gemcitabine | 1 |
| Irinotecan | 1 |
| Methotrexate | 1 |
| Nivolumab | 2 |
| Panitumumab | 1 |
| Pembrolizumab | 1 |
| Radiotherapy | 1 |
| Radiotherapy and cetuximab | 1 |
| Radiotherapy and cisplatine | 1 |
| Raltitrexed | 1 |
| Raltitrexed and irinotecan | 2 |
| Raltitrexed and oxaliplatine | 1 |
| Taxane | 2 |
| Taxane and cetuximab | 1 |
| Taxane and cisplatine | 4 |

Figure S1. Time from first (panel A) and last (most recent, panel B) fluoropyrimidine dose administration and cardiovascular event



Boxes are median and interquartile range, plots are 5-95%
